# Comparative Genomics of *Clostridium perfringens* Reveals Patterns of Host-Associated Phylogenetic Clades and Virulence Factors

**DOI:** 10.3389/fmicb.2021.649953

**Published:** 2021-06-09

**Authors:** Renae R. Geier, Thomas G. Rehberger, Alexandra H. Smith

**Affiliations:** Arm and Hammer Animal and Food Production, Church & Dwight Co., Inc., Waukesha, WI, United States

**Keywords:** *Clostridium perfringens*, necrotic enteritis, hemorrhagic bowel syndrome, avian, ruminant

## Abstract

*Clostridium perfringens* is an opportunistic pathogenic bacterium that infects both animals and humans. *Clostridium perfringens* genomes encode a diverse array of toxins and virulence proteins, which continues to expand as more genomes are sequenced. In this study, the genomes of 44 *C. perfringens* strains isolated from intestinal sections of diseased cattle and from broiler chickens from diseased and healthy flocks were sequenced. These newly assembled genomes were compared to 141 publicly available *C. perfringens* genome assemblies, by aligning known toxin and virulence protein sequences in the assemblies using BLASTp. The genes for alpha toxin, collagenase, a sialidase (*nanH*), and alpha-clostripain were present in at least 99% of assemblies analyzed. In contrast, beta toxin, epsilon toxin, iota toxin, and binary enterotoxin of toxinotypes B, C, D, and E were present in less than 5% of assemblies analyzed. Additional sequence variants of beta2 toxin were detected, some of which were missing the leader or signal peptide sequences and therefore likely not secreted. Some pore-forming toxins involved in intestinal diseases were host-associated, the *netB* gene was only found in avian isolates, while *netE*, *netF*, and *netG* were only present in canine and equine isolates. Alveolysin was positively associated with canine and equine strains and only present in a single monophyletic clade. Strains from ruminant were not associated with known virulence factors and, except for the food poisoning associated clade, were present across the phylogenetic diversity identified to date for *C. perfringens*. Many *C. perfringens* strains associated with food poisoning lacked the genes for hyaluronidases and sialidases, important for attaching to and digesting complex carbohydrates found in animal tissues. Overall, the diversity of virulence factors in *C. perfringens* makes these species capable of causing disease in a wide variety of hosts and niches.

## Introduction

*Clostridium perfringens* is a Gram-positive facultative anaerobic bacterium that is a normal inhabitant of the soil as well as the gastrointestinal tracts of healthy animals. However, *C. perfringens* is also an opportunistic pathogen known for its ability to cause gas gangrene/clostridial myonecrosis of the skin (Buboltz and Murphy-Lavoie, 2020) as well as food-poisoning in humans that costs the United States approximately $343 million annually (ERS-USDA, 2014). In cattle, it can cause hemorrhagic bowel syndrome (HBS), enterotoxaemia, and abomastitis (Nowell et al., 2012; USDA, 2018; Diancourt et al., 2019). In poultry, it causes necrotic enteritis (NE), a disease that has seen an increase with decreased antibiotic use in the poultry industry and results in approximately 2 billion US dollars globally in losses annually (Van der Sluis, 2000). Other enteric diseases in which *C. perfringens* are implicated are canine acute hemorrhagic diarrhea syndrome (AHDS) and foal necrotizing enteritis (FNE; Gohari et al., 2015).

The first *C. perfringens* genome sequence, published in 2002, greatly expanded our understanding of the vast array of virulence genes and toxins (Shimizu et al., 2002). In 2018, the toxin typing scheme for *C. perfringens* was expanded to include the pore-forming toxin, NetB, shown to be relevant to NE (Rood et al., 2018). The toxinotyping scheme is based on the presence of alpha toxin, beta toxin, epsilon toxin, iota toxin, enterotoxin, and NetB toxin. These toxins are used for typing but are not the only factors important to disease as *C. perfringens* is known to produce multiple additional toxins and virulence factors (Revitt-Mills et al., 2015; Kiu and Hall, 2018).

Alpha toxin is the most conserved and well-known toxin. It hydrolyzes membrane phospholipids (lecithin, phosphatidylcholine, and sphingomyelin) in blood, skin, and muscle cells, and it often acts synergistically with other toxins (MacFarlane and Knight, 1941; Titball, 1993; Songer, 1996; Awad et al., 2001; Hickey et al., 2008; Uzal et al., 2010). Recent reviews summarize the knowledge on the pore-forming toxins (beta toxin, beta2 toxin, epsilon toxin, enterotoxin, NetB, NetE, NetF, NetG, perfringolysin, and alveolysin) and intracellular toxins which break down the actin cytoskeleton (iota toxin) or disrupt cell signaling (TpeL toxin; Kiu and Hall, 2018; Uzal et al., 2018).

There are other enzymes that may not be essential for disease but contribute to virulence. Some of these are proteases that degrade proteins into available forms of amino acids. *Clostridium perfringens* is unable to *de novo* synthesize many amino acids and thus, must obtain them from the environment (Sebald and Costilow, 1975; Shimizu et al., 2002). These proteases are likely important for degradation of host tissue which enables *C. perfringens* to both obtain nutrients and facilitate toxin diffusion (Matsushita et al., 1994; Awad et al., 2000). Carbohydrate-active enzymes (CAZymes) are also important for the virulence of *C. perfringens*. For instance, the release of sialic acid by *C. perfringens* sialidases has been shown to increase the activity of toxins (alpha and epsilon), increase adhesion to host cells by altering the charge of the cell surface, and can be used as a carbon source (Severi et al., 2007; Almagro-Moreno and Boyd, 2009; Chiarezza et al., 2009; Li et al., 2011, 2016; Therit et al., 2015; Juge et al., 2016; McClane and Shrestha, 2020; Wang, 2020).

Previous genome sequencing studies of clinical *C. perfringens* strains from equine, canine, and poultry have revealed specific host-associated virulence factors (Gohari et al., 2017; Lacey et al., 2018). Strains from human, food, environmental, and ruminant sources were also included in comparative genome studies (Kiu et al., 2017; Lacey et al., 2018), however, only four ruminant isolates were available in public databases. To increase the diversity of sequenced *C. perfringens* genomes and improve our understanding of potential host-related virulence factors, 22 *C. perfringens* isolated from healthy and diseased poultry flocks and 22 *C. perfringens* isolated from dairy cow intestinal tracts with HBS were sequenced. These genomes were compared to 141 publicly available genomes and analyzed for the presence of the major known virulence factors to ascertain associations with host and diseases and determine evolutionary relationships.

## Materials and Methods

### Strain Isolation

Intestinal tracts or fecal samples were obtained in the United States from commercial broiler operations and dairy farms. All animal facilities were operated under the standards for humane care and treatment for commercial animals set in the Animal Welfare Act (AWA; USDA, 2020) and the National Dairy Farmers Assuring Responsible Management animal care program (National Milk Producers Federation Board of Directors, 2019). Live broilers were obtained from flocks during NE outbreaks and from healthy flocks. The broilers were sacrificed on farm by cervical dislocation in accordance with the integrator’s animal welfare practices. The gastrointestinal tracts from the duodenal loop to the cloaca were removed, placed into sterile Whirl-pak® bags (B01297, Nasco, Fort Atkinson, WI, United States), and sent to the laboratory in Waukesha, WI overnight, on ice. For each broiler, 6 cm sections of the duodenum, jejunum, and ileum were dissected, and luminal contents removed by rinsing with sterile 0.1% peptone (Bacto™ Peptone, Becton, Dickinson and Company, Sparks, MD, United States). The three sections from each bird were combined in a filtered Whirl-pak® bag (B01348, Nasco, Fort Atkinson, WI, United States). Fecal grabs or the infected portion of the gastrointestinal tract (discoloration, blood clotting within the jejunum) from cows that had suffered a digestive death were collected within 6 h of death. The samples were placed in a zip-top freezer bags and sent to the laboratory in Waukesha, WI overnight, on ice.

Intestinal tracts or fecal samples were diluted 1:9 with sterile 0.1% peptone and masticated at 300 rpm, for 1 min in a Stomacher (Model 400 circulator, Seward, England). Serial dilutions prepared from the filtered side of the Whirl-pak® bags were pour-plated in duplicate with tryptose sulfite cycloserine (TSC) agar (Thermo Fisher Scientific, Waltham, MA, United States), and incubated at 37°C with anaerobic gas packs (R681001, Remel, Lenexa, KS) overnight. Up to 20 representative isolates per sample were grown in Reinforced Clostridial Medium (Thermo Fisher Scientific, Waltham, MA, United States) before storage at −80°C.

### Strain Selection

In general, one isolate per animal was included and isolates from the same animal were included only if they produced differential randomly amplified polymorphic DNA (RAPD) typing banding patterns (Power, 1996). Primers and PCR conditions were as described previously (Baker et al., 2010) with the only difference being that amplicon fragments were separated on a 5300 Fragment Analyzer System (Agilent, Santa Clara, CA, United States). Nineteen *C. perfringens* isolates were from broiler chicken intestinal samples collected during NE outbreaks, however, the presence of NE lesions was not recorded for these intestinal tracts, while three isolates were from healthy broiler chicken intestinal samples. Twenty-one isolates were from dairy cow intestinal samples with HBS, and one isolate was from a fecal sample of a dairy cow with HBS.

### Genome Sequencing and Assembly

RNA-free DNA was isolated using a phenol-chloroform method with RNase treatment and precipitated with ethanol. Genomic DNA integrity was evaluated on a 0.75% agarose gel and quantified using Qubit (Thermo Fisher Scientific, Waltham, MA, United States). The 16S rRNA gene was PCR amplified and Sanger sequenced to confirm identity. Shotgun libraries were prepared with Nextera Flex kits (Illumina, San Diego, CA, United States) and sequenced for 251 cycles from each end on a MiSeq using a MiSeq 500-cycle sequencing kit v3 (Illumina, San Diego, CA, United States) or sequenced for 151 cycles from both ends on an iSeq 100 using iSeq 100 i1 Reagent (Illumina, San Diego, CA, United States). For some genomes, shotgun libraries were prepared with Hyper Library Construction Kit (Kapa Biosystems, Wilmington, MA, United States) and sequenced for 300 cycles from each end on a MiSeq using a MiSeq 600-cycle sequencing kit v3 (Illumina, San Diego, CA, United States). All reads were demultiplexed using bcl2fastq Conversion Software (Illumina, San Diego, CA, United States). Draft genome assemblies were generated using SPAdes 3.13.1 using default parameters (Bankevich et al., 2012). Reads were aligned to genome assemblies with bwa mem v0.7.17 (Li and Durbin, 2009). Bam files were converted to sam files with samtools, and coverage was calculated using bedtools (Quinlan and Hall, 2010).

### Bioinformatic Analysis

Draft genome assemblies were compared with all 141 publicly available *C. perfringens* genome assemblies from NCBI RefSeq as of February 25, 2020. All available metadata for genomes were collected and host information was categorized into relevant groups to improve statistical power (e.g., chicken and turkey were classified as avian; Supplementary Table 1). Taxonomy of genome assemblies was confirmed by aligning the 16S ribosomal RNA gene sequence to the 16S database in NCBI. Assembly statistics (Supplementary Table 1) were generated using QUAST v5.0.2 (Gurevich et al., 2013). A maximum likelihood tree was generated by performing SNP calling on genome assemblies with CSI Phylogeny using the reference strain *C. perfringens* ATCC 13124 (Kaas et al., 2014). The phylogenetic tree was visualized and annotated using iTol v5.6.2 (Letunic and Bork, 2016). Genomes were annotated using Prokka v1.14.6 (Seemann, 2014). A BLAST protein database was made from virulence factor protein sequences (Supplementary Table 2) using makeblastdb (BLAST+ v2.9.0). Prokka protein annotations were aligned to protein databases using BLASTp (BLAST+ v.2.9.0, -evalue 1 -max_target_seqs 1 -qcov_hsp_perc 50; Camacho et al., 2009). Both consensus and atypical variants of beta2 were used. These parameters set a threshold of 50% alignment length, which is appropriate for draft genome assemblies to reduce false negatives. We chose a threshold of 80% identity to allow for the detection of variants. For known variants (PfoA-Alv and NetB-NetF), we increased the percent identity threshold to 90% to distinguish between these closely related proteins. A binary matrix of virulence gene presence or absence was created from the BLASTp results. Beta2 protein sequences were analyzed for signal peptide content using SignalP v5 (Armenteros et al., 2019) and aligned with Clustal Omega v1.2.4 (Sievers et al., 2011). *In silico* PCRs of previously published beta2 primers were done using the -search_pcr function of USEARCH v10.0.240 with the following settings -strand both -maxdiffs 2 -minamp 30 -maxamp 2000 (Edgar, 2010).

The virulence gene presence within a category and the associated lift (Tufféry, 2011) was computed for each category. Lift is common measure in data mining algorithms to identify the strength for pairwise association of outcomes or even possibly sets of outcomes where outcomes are defined in terms of presence or absence. The lift is defined as the rate of joint occurrence of the pair of outcomes/sets of outcomes in the dataset relative to the product of the rate of each outcome, i.e., for outcomes X and Y, lift = Prob(X & Y)/[Prob(X)*Prob(Y)]. The lift provides an indication of the relative magnitude of presence or absence of the gene within a category as compared to the presence across all isolates. Lift values greater than 1 indicate a higher presence in the category compared to the presence in all strains and, conversely, lift values less than indicate lower prevalence in the category. A 2 × 2 contingency table was created for each virulence gene (presence/absent) and category (yes/no by strain) and tested for significant association using Fisher’s Exact test for independence (Agresti, 2002). A Bonferroni adjustment was implemented to provide an overall 0.05 error rate across all comparisons. All computations were performed using R version 3.5.0.

## Results and Discussion

### Overview of *C. perfringens* Genome Assemblies

Between 199,762 and 3,020,471 paired reads were generated for each of the 44 strains sequenced resulting in a range of 23- and 433-fold coverage for each strain (Supplementary Table 3). Assembly statistics were generated using QUAST, and the number of coding sequences was counted from Prokka annotations (Supplementary Table 1). The minimum and maximum number of contigs, total length, and percent GC, N_50_, and L_50_ all fell within the range for RefSeq strains, except the length of strain CHD30685R, which was 33 kb shorter than the shortest RefSeq assembly.

The isolate metadata are shown in Supplementary Table 1. The largest group was of avian strains (*n* = 61) which were all chicken associated, except for one strain isolated from a turkey, with 49 of these strains from flocks experiencing NE. A total of 34 isolates were isolated from ruminants: 25 from cattle, four from lamb and sheep, four from llamas, and one strain was isolated from a bison. The NCBI database contained 29 human-associated strains, most (*n* = 12) of which had no known disease associations, while the rest were from healthy humans (*n* = 5), necrotizing enterocolitis (*n* = 3), food poisoning (*n* = 5), gas gangrene (*n* = 2), diarrhea (*n* = 1), necrotizing enteritis (NCTC8081; Deguchi et al., 2009), and an ICU patient (*n* = 1). There were 17 canine isolates, 16 of which were isolated from canine AHDS. The 16 equine isolates were all isolates from FNE. The 15 food-associated isolates have very little disease information deposited with them but are likely food poisoning strains. Lastly, five environmental isolates from river water, soil, or sludge, three porcine intestinal disease-associated isolates, and one mouse isolate were downloaded from the NCBI database. Four of the strains had no host or disease metadata.

### Toxins and Virulence Factors

The toxin and virulence factor profiles were determined using BLASTp for all 185 *C. perfringens* strains used in the analysis (Supplementary Table 1). The prevalence of each gene varied from less than 1 to 100% (Table 1). Alpha toxin (*plc*), collagenase (*colA*), the small intracellular sialidase (*nanH*), and alpha-clostripain (*ccp*) presence were highly conserved and were present in at least 99% of assemblies analyzed. All 185 alpha toxin protein sequences were at least 96% identical to the type strain ATCC 13124, although it should be noted that the JFP992 sequence was split over two contigs and the predicted alpha toxin protein sequence for UDE_95-1372 was truncated at the N-terminus. Very few strains encoded beta toxin (3%), epsilon toxin (3%), and iota toxin (2%).

**Table 1:** Prevalence of toxin and virulence genes in 185 *Clostridium perfringens* genomes together with information on location [chromosome (C) or a plasmid (P)] as well as the type of protein encoded [membrane-damaging phospholipase (PLC), pore-forming toxin (PFT), intracellular toxin (I), protease (P), or carbohydrate-active enzyme (CAZyme)].

Name	Gene	Location	Type	Strains	%
Alpha toxin	*plc*	C	PLC	185	100.0%
Alpha-clostripain	*ccp*	C	P	185	100.0%
Sialidase	*nanH*	C	CA	184	99.5%
Collagenase	*colA*	C	P	183	98.9%
Hyaluronidase	*nagH*	C	CA	161	87.0%
Sialidase	*nanI*	C	CA	160	86.5%
Sialidase	*nanJ*	C	CA	159	85.9%
Hyaluronidase	*nagJ*	C	CA	156	84.3%
Hyaluronidase	*nagI*	C	CA	154	83.2%
Perfringolysin	*pfoA*	C	PFT	151	81.6%
Hyaluronidase	*nagK*	C	CA	140	75.7%
Hyaluronidase	*nagL*	C	CA	115	62.2%
Beta2 toxin	*cpb2*	P	PFT	109	58.9%
Enterotoxin	*cpe*	C/P	PFT	56	30.3%
NetB toxin	*netB*	P	PFT	38	20.5%
Alveolysin	*alv*	C	PFT	35	18.9%
NetE toxin	*netE*	P	PFT	28	15.1%
NetF toxin	*netF*	P	PFT	28	15.1%
Large cytotoxin	*tpeL*	C/P	I	19	10.3%
NetG toxin	*netG*	P	PFT	16	8.6%
Beta toxin	*cpb*	P	PFT	6	3.2%
Epsilon toxin	*etx*	P	PFT	5	2.7%
Iota toxin	*iap & ibp*	P	I	4	2.2%
Binary enterotoxin	*becA & becB*	P	I	1	0.5%

### Toxinotypes

We classified the strains into toxinotypes using the BLASTp toxin profiles. Approximately, 94% of the strains analyzed were type A, F, or G (Table 2). Toxinotype A encodes alpha toxin while the other typing toxins, other than *cpe* in some strains, are all plasmid encoded. Toxinotype A strains comprised 43.8% of the strains and were present in all host categories. Toxinotype F strains encode enterotoxin (*cpe*) either on the chromosome or plasmids and were the predominant toxinotype in isolates from canine, equine, and food. One avian strain and seven of the 29 human isolates were also Toxinotype F. Toxinotype G strains encode *netB*, which is plasmid-borne (Lepp et al., 2010, 2013) and was only present in avian isolates and in 76% of the NE associated isolates. The NetB pore forms in chicken hepatocytes and red blood cells of duck, chicken, and goose, and is important for the development of NE (Keyburn et al., 2008, 2010; Yan et al., 2013; Lacey et al., 2018; Yang et al., 2019b). Based on epidemiological data, there is debate in the literature whether NetB is necessary to cause NE (Martin and Smyth, 2009; Tolooe et al., 2011; Yang et al., 2018). In a challenge model, two of three *netB* positive strains produced disease at a high rate (79–89%), but a *netB* negative strain still affected 44% of challenged birds (Cooper and Songer, 2010). A necrotic enteritis induction model would be necessary to determine if the 12 NE associated strains that did not encode *netB* are commensals or can cause disease.

**Table 2:** Toxinotypes of 185 *C. perfringens* genomes based on the typing scheme of Rood et al. (2018).

Toxinotype	Alpha toxin (*plc*)	Beta toxin (*cpb*)	Epsilon toxin (*etx*)	Iota toxin (*iap* and *iab*)	Enterotoxin (*cpe*)	NetB (*netB*)	Strains	%
A	+	−	−	−	−	−	81	43.8%
B	+	+	+	−	−	−	1	0.5%
C	+	+	−	−	±	−	5	2.7%
D	+	−	+	−	±	−	4	2.2%
E	+	−	−	+	±	−	4	2.2%
F	+	−	−	−	+	−	52	28.1%
G	+	−	−	−	−	+	38	20.5%

Strains of Toxinotypes B, C, D, and E only made up 3% or fewer of the total strains analyzed. These toxinotypes are acknowledged to be associated with many livestock diseases (Songer, 1996; Billington et al., 1998; Filho et al., 2009; Munday et al., 2019) and are incorporated into veterinary vaccines (Ferreira et al., 2016, 2018), and yet very few have been sequenced.

### Beta2 Toxin Variants

**Figure 1:**
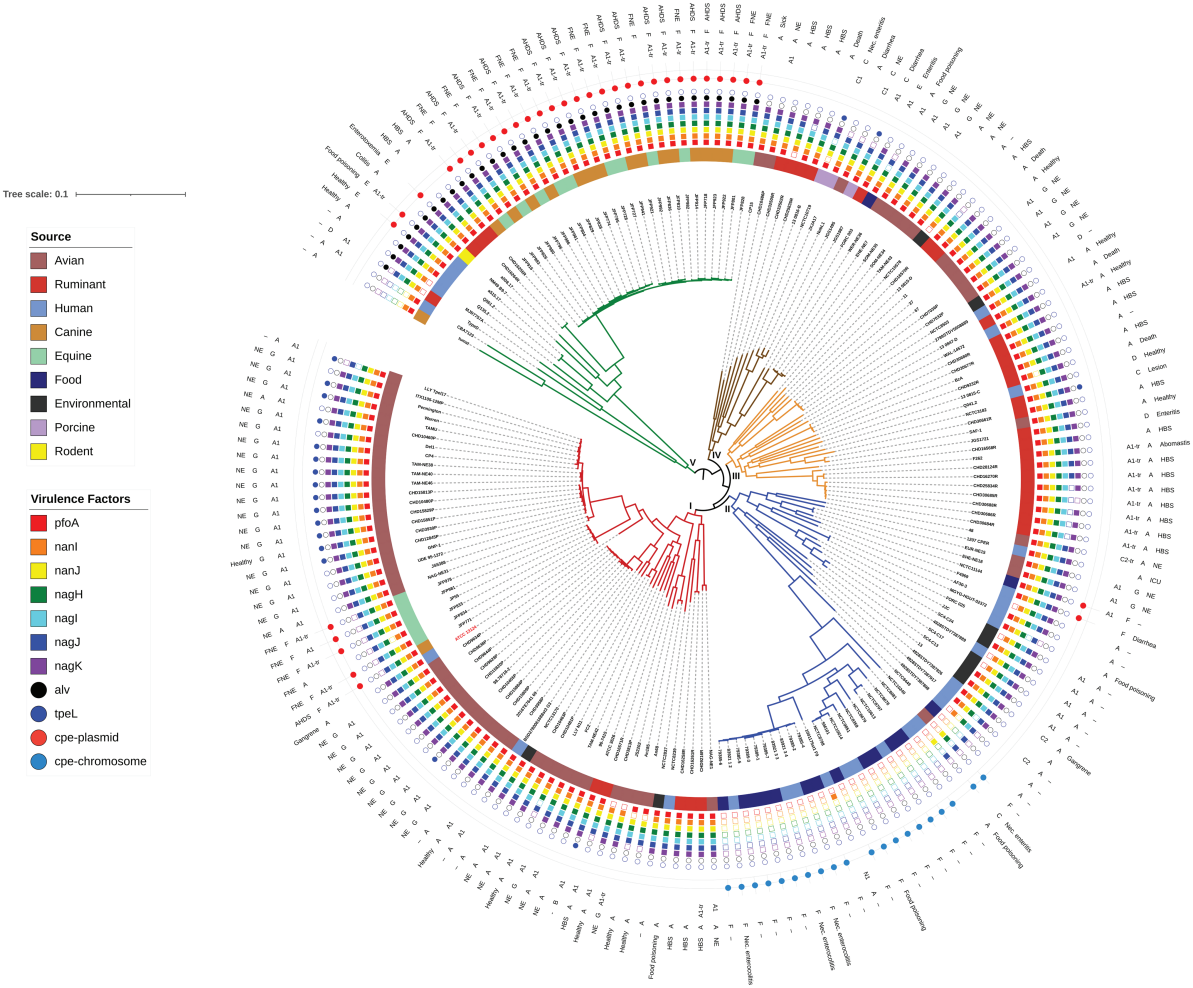
Maximum likelihood phylogenetic tree of *C. perfringens* genome assemblies determined by single nucleotide polymorphisms compared to the reference strain ATCC 13124. The reference strain and Clade I are shown in red. Clade II is the food poisoning associated clade and Clade V is the alveolysin clade. Host associations are shown on the inner ring, followed by specific virulence factors, the outer three rings indicate the beta2 variant, toxinotype, and health or disease association (if known) of each strain. The tree is rooted at the midpoint.

**Figure 2:**
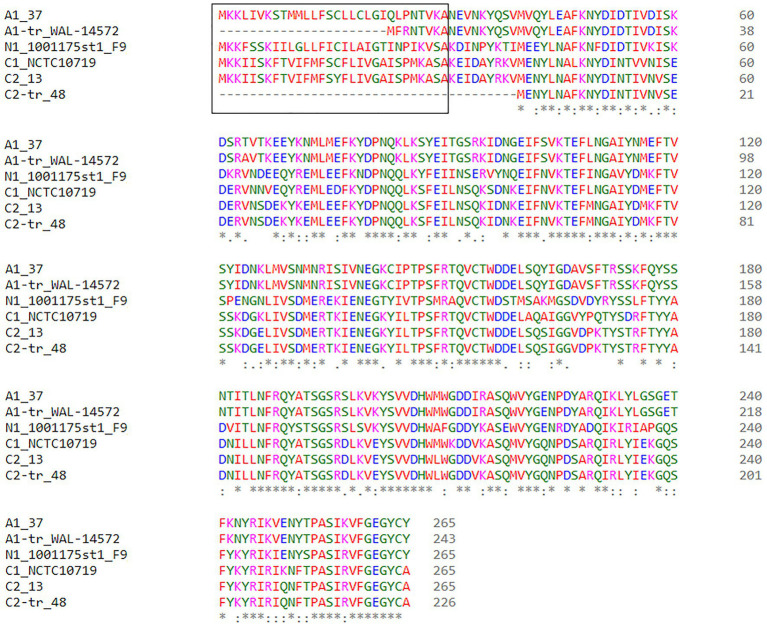
Beta2 (*cpb2*) toxin clustal alignment of each representative beta2 protein type. Amino acids are colored based on their physical properties: blue (acidic), red [small hydrophobic (includes aromatic except Y)], magenta (basic), and green [other (polar, Y)]. The signal peptide sequences are shown in the black box.

Beta2 toxin is cytotoxic for intestinal cells and there is a strong association between *C. perfringens* strains that encode *cpb2* and gastrointestinal diseases in pigs, although there are at least two variants of the beta2 toxin and this diversity is not always acknowledged (Gibert et al., 1997; Garmory et al., 2000; Waters et al., 2003; Fisher et al., 2005; Jost et al., 2005). To investigate the sequence variation between the consensus and atypical genes, as well as signal peptide variation, we classified the beta2 toxin sequences by amino acid identity and signal peptide content. After combining the results of the consensus and atypical beta2 toxin BLASTp results, *cpb2* was identified in 109/185 (59%) of strains analyzed, and one strain, JGS 1495, had both the consensus and atypical variants located on different contigs. Six types of beta2 were identified: five that have been described [three consensus (C) types and two atypical (A) types] and one novel type that we designated N1. Only one strain encoded the N1 type, 1001175st1_F9, a strain isolated from healthy human stool (Yang et al., 2019a). The consensus variant was divided into two types, C1 and C2, which are ~92% identical at the protein level. We further classified the beta2 sequences by signal peptide content and added a -tr designation in Figure 1 for those strains lacking a signal peptide. Of the six consensus *cpb2*, two were the original consensus variant, C1, two were the C2 variant described in a 2005 publication (Fisher et al., 2005), and one was a C2-tr variant. Of the atypical beta2 toxin sequences, which are approximately 63% identical to the consensus variant, 64 (62%) were A1 and 39 (38%) were A1-tr. A representative from each of these six variants was selected for protein sequence alignment (Figure 2).

Beta2 toxin disease associations are often of a specific type and the presence of a signal peptide may play an important functional role and it is therefore important to acknowledge in disease association studies. We performed *in silico* PCR to determine which types would have been detected in various publications (Supplementary Table 4). The PCR protocol in the original Cpb2 paper used to associate *cpb2* with intestinal disease in horses and piglets would have only detected the C1 type (Gibert et al., 1997). Similarly studies associating *cpb2* and diarrhea in piglets would also have detected the C1 type (Waters et al., 2003). In addition, there is an association between Cpb2 and autism spectrum disorder, and these studies used primers that also would have detected the C1, C2, and C2-tr types, but not the atypical variants or the novel variant N1 (Garmory et al., 2000; Alshammari et al., 2020). In a study by Kircanski et al. (2012), when Cpb2 protein levels were quantified in culture supernatants by Western blot, 95% of consensus isolates and 75% of atypical isolates were shown to express the protein. The study would have successfully identified C1, A1 (and A1-tr), and possibly identified C2 (and C2-tr). They would have been able to distinguish between consensus and atypical variants but would not have been able to distinguish the presence of the signal peptide potentially explaining why 25% of atypical *C. perfringens* and 5% of consensus isolates did not express beta2 toxin.

Of the three strains in the present study which were porcine associated, one encoded a C1 type (NCTC 10719), one encoded both a C1 and an A1 type (JGS 1495), and one lacked beta2 toxin (JXJA17). Ruminant, canine, and equine assemblies encode only atypical (primarily A1-tr) beta2 toxin. Chicken isolates primarily encoded the A1 type. In the first paper describing the atypical variant, Jost et al. (2005) noticed a similar pattern that atypical isolates were more often identified in *C. perfringens* strains that were isolated from livestock other than pigs and not expressed.

These findings add a new dimension to this previous research which reveals that the associations are often of a specific Cpb2 type. Future studies which take this variation of sequence and signal peptide content into account are likely to see stronger associations between Cpb2 and various diseases.

### *Clostridium perfringens* Phylogeny

Phylogenetic relationships between the strains were determined by CSI Phylogeny (Kaas et al., 2014) which analyzes the SNPs across reads using a reference genome. *Clostridium perfringens* ATCC 13124 with 3,256,683 nucleotides was used as the reference strain. The percentage of the reference genome that was covered by all isolates was 48.06% with 1,565,015 positions found in all 185 genomes. The maximum likelihood tree generated is shown in Figure 1, with five clades labeled I through V. The reference strain, ATCC 13124 was present in Clade I with 60 strains which contained 43 of the 61 avian isolates, five equines, five ruminants, three humans, one canine, and two environmental isolates.

### Alveolysin Clade

Alvoelysin (*alv*) was the only toxin limited to a single clade as all 35 strains that encoded *alv* were present in Clade V confirming a previous study showing it was clade-specific (Kiu et al., 2019). Alveolysin is an understudied toxin of *C. perfringens* that is similar to perfringolysin (Kiu et al., 2019) with both being cholesterol-dependent cytolysins, previously known as thiol-activated cytolysins (Billington et al., 2000). Gene duplications are frequent mutations in microbes (Reams and Roth, 2015), and we therefore hypothesize that alveolysin may have arisen from a gene duplication of perfringolysin followed by divergence during evolution as the two toxins are similar (~79% similarity) and generally encoded as little as 5 kb apart, although lateral gene transfer cannot be ruled out.

Within Clade V is a sub-clade of 26 strains that contains clinical isolates associated with canine AHDS and FNE that are all type F. These strains appear almost clonal, but not only were they isolated from different host species but also across multiple outbreaks between 1999 and 2014 in three different countries (Gohari et al., 2017). Of the 26 strains in the sub-clade, 23 encoded *netE* and *netF* (88%). NetF toxin is very similar in structure to NetB, but it has only been identified in isolates from canine AHDS and FNE (Gohari et al., 2015, 2016, 2017).

Also, of interest within Clade V is that three of the four toxinotype E strains (Q061.2, a515.17, and a508.17) are present. These strains contain a variant iota toxin which is 84–87% similar to the typical iota toxin sequence. The other toxinotype E strain (JGS 1987) is outside this clade and has the typical iota toxin sequence. This iota toxin variant has been identified in other *C. perfringens* that lack public genome assemblies (PB-1, 3441, TGII002, and TGII003; Miyamoto et al., 2011). The strains in that study and each of the three variant strains in the present study also have a variant enterotoxin protein sequence (~96% similar to the other 53 sequences) located on the same plasmid as the iota toxin genes indicating evolutionary divergence of the plasmid within this clade. Further studies to obtain complete plasmid sequences need to be done to validate this supposition.

The only strain to encode binary enterotoxin (*becA* and *becB*), Q135.2 (IQ3), is also in clade V and was isolated from a fecal sample obtained from a healthy child (Kiu et al., 2019). The *becA* and *becB* genes are plasmid-encoded and seem to be rare (Kiu et al., 2019; Matsuda et al., 2019).

Further research is needed of the virulence potential of the strains in Clade V due to the presence of alveolysin, an understudied chromosomal toxin as well as several variant and rare toxins carried on plasmids.

### Food Poisoning Associated Clade

Fourteen of the 15 strains isolated from foods were present in Clade II. Seventeen of the human isolates, three from food poisoning cases and three from necrotizing enterocolitis were also present in Clade II, as were five environmental and five avian isolates. The 20 strains in which chromosomal *cpe* genes were detected were present in a sub-clade of 27 strains. Ten of these isolates from both food and humans appear clonal and were submitted in the same bioproject (PRJNA436899) and are therefore most likely from the same clinical outbreak. Experimental evidence suggest that strains carrying chromosomal *cpe* are more heat-tolerant allowing them to survive better if food is undercooked (Sarker et al., 2000). Our results confirm a previous study where strains that carry *cpe* chromosomally are related and that they lack the *pfoA* gene (Deguchi et al., 2009). The majority (23) of these 27 strains also lacked the hyaluronidase and sialidases that enhance a strains ability to colonize the intestinal tract (Navarro et al., 2018).

The alpha toxin protein sequences in the sub-clade of 27 were divergent with less than 97% similarity to the sequence from the type strain, ATCC 13124. The alpha toxin gene is located near the origin of replication, which is evidence of its importance as it is the first area to be replicated during cell division and is generally highly conserved, thus genetic changes in it are likely to reflect evolution (Rood and Cole, 1991; Uzal et al., 2010). This chromosomal variation indicates that these strains form a distinct evolutionary lineage which may be less adapted to the host environment and more opportunistic than other strains. Although necrotizing enterocolitis is not associated with food-poisoning, the disease most often occurs in premature infants with immature gastrointestinal tract microbiota. They appear to be more likely to be transiently present in the gastrointestinal tract, whereas the host-adapted strains cause more lethal diseases in adult animals.

### Host and Environmental Associations

**Figure 3:**
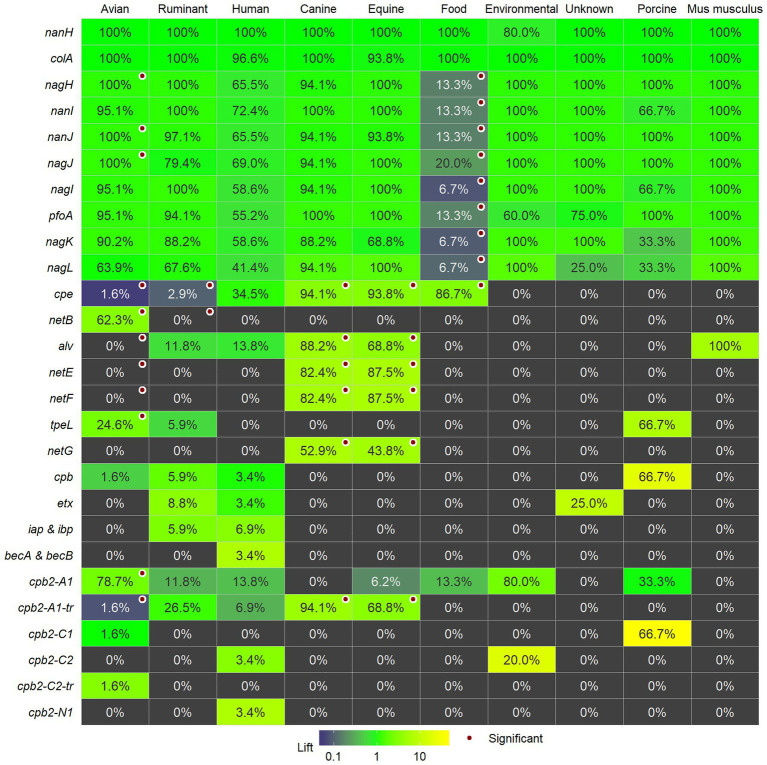
The percentage of isolates within each category for each of the individual toxins and virulence factors and whether they are significantly associated with the category are marked with a red circle. The number of strains per category is as follows: avian (61); ruminant (34); human (29); canine (17); equine (16) food (15), and environmental strains (5). Unknown (4), porcine strains (3), and mouse (1) are shown, but were not evaluated for associations. Cells are colored by lift: values greater than 1 indicate a higher presence in the category compared to the presence in all strains and, conversely, lift values less than indicate lower prevalence in the category.

We determined significant associations of virulence genes with categorical host metadata using Fisher’s Exact test for independence and this data are shown in Figure 3 together with the lift that provides an indication of the relative magnitude of presence or absence of the gene within a category as compared to the presence across all isolates. In comparing avian strains (*n* = 61) to the other categories of isolates, there was a significantly higher proportion of isolates with *netB*, *cpb2-A1*, *tpeL*, *nanJ*, *nagJ*, and *nagH*. Avian strains showed lower frequencies of *cpb2-A1-tr*, *cpe*, *netE*, *netF*, and *alv*. Ruminant strains (*n* = 34) showed lower prevalence for *netB* and *cpe*. Canine strains (*n* = 17) and equine strains (*n* = 16) showed higher prevalance for *alv*, *cpb2-A1-tr*, *cpe*, *netE*, *netF*, and *netG*. Food (possibly food poisoning) strains were positively associated with *cpe* and showed lower prevalence for *pfoA*, two sialidases (*nanI*, *nanJ*), and four hyaluronidases (*nagH*, *nagI*, *nagK*, and *nagL*). There were no significant associations for genes and human strains (*n* = 29) and environmental strains (*n* = 5). Unknown (*n* = 4), porcine strains (*n* = 3), and mouse (*n* = 1) were not evaluated for associations.

Our results confirmed previous data that *netB* is associated with avian strains (Keyburn et al., 2008, 2010; Lepp et al., 2010, 2013; Lacey et al., 2018). We only found one report of *netB* being detected in species other than poultry and that was in a ruminant isolate (Martin and Smyth, 2009). The other toxin gene associated with poultry is *tpeL*, which was also detected in ruminant and porcine strains. TpeL glycosylates cell signaling proteins resulting in apoptosis (Guttenberg et al., 2012; Schorch et al., 2014; Nagahama et al., 2015) and have been shown to be responsible for increased NE pathogenicity (Coursodon et al., 2012; Shojadoost et al., 2012; Gu et al., 2019). Our data indicate that the A1 beta2 toxin variant with the signal peptide is associated with avian strains, although this variant is detected in other host strains too.

As in previous reports, the pore-forming toxins, *netE*, *netF*, and *netG*, are associated with canine and equine strains (Gohari et al., 2016, 2017, 2020; Sindern et al., 2019) and these toxins were not detected in any other strains. These canine and equine strains are unique among the diversity of strains from other hosts and environments. They are present in Clades I and V and related strains appear almost clonal even though they are from distinct hosts and from epidemiologically unrelated clinical isolates collected from the United States, Canada, and Switzerland between 1999 and 2014 (Gohari et al., 2017). Plasmid-borne enterotoxin was present across both clades, and the predominant beta2 variant in these strains was the A1 variant without the signal peptide. Alveolysin, associated with equine and canine strains, was also present in strains from other hosts in Clade V. Challenge assays either *in vitro* or *in vivo* may reveal what it is about these strains or the two hosts that cause an almost clonal population to be present across countries and disease outbreaks.

There were no positive associations with any of the investigated toxins or virulence factors with strains from ruminants. Ruminant strains were defined by the absence of enterotoxin and *netB* genes. Previous experimental induction of disease in a calf ileal loop model indicated that diverse *C. perfringens* strains from ruminant, chicken, and human origins could cause necrohaemorrhagic lesions (Valgaeren et al., 2013), and alpha and perfringolysin toxin were sufficient to cause lesions in this model (Verherstraeten et al., 2013). Novel toxin genes were not detected in the genome of a bovine clostridial abomasitis isolate strain F262, however, the strain did produce perfringolysin O, alpha-toxin, and beta2-toxin (Nowell et al., 2012). *Clostridium perfringens* Type D are associated with ruminant enterotoxaemia, mostly in lambs, but also in sheep and goats (Popoff, 2011), however, epsilon toxin was not commonly present in the sequenced genomes. To date, therefore, no specific toxins or virulence factors are associated with the 26 sequenced clinical *C. perfringens* strains of ruminant origin, however, it may be dependent on the type of disease, and the 22 strains sequenced in this study were all associated with HBS. There is genetic diversity in the strains from ruminants as they are present in all clades except for Clade II, however, 20 of the 32 ruminant strains were present in Clade III. Therefore, further analysis of these genomes may reveal genes promoting colonization or growth in the intestine that could affect pathogenesis in ruminants.

Our results have confirmed previous data that certain toxin genes are host-associated such as *netB* in avian strains (Keyburn et al., 2008, 2010; Lepp et al., 2010, 2013; Lacey et al., 2018) and *netE*, *netF*, and *netG* in canine and equine strains (Gohari et al., 2016, 2017, 2020; Sindern et al., 2019). In addition, our data indicate that there are differences in beta2 toxin variants between hosts with the A1 variant with the signal peptide being associated with avian strains and the A1 variant without the signal peptide associated with canine and equine strain. However, considering the role that *C. perfringens* has in multiple livestock and human diseases there is still limited data on the virulence factors and host specificity of these pathogens. *Clostridium perfringens* are found in a wide variety of hosts and environments; however, most of the strains selected for study and genome sequencing are associated with a handful of diseases and may not represent the diversity present in both hosts and environment. More specifically, few strains acknowledged to be associated with livestock diseases, such as Types B, C, D, and E have been sequenced. Vaccination efforts for livestock have focused on these toxinotypes (Ferreira et al., 2016) which may be why they are absent from recent studies, however, strains should be present in culture collections that could be sequenced to aid in understanding this pathogen. A better understanding of this opportunistic pathogen that is a member of the gut microbiota can lead to more targeted preventative measures to reduce factors that can lead to overgrowth and clinical diseases.

## Conclusion

This is the most comprehensive comparative genomics study of *C. perfringens* virulence factors to date. Only four of the 24 virulence factors were highly conserved and were present in at least 99% of assemblies analyzed. Types A, F, and G represent 93% of sequenced isolates, while Type B, C, D, and E are underrepresented in publicly available genome sequences even though they are associated with many livestock diseases. The sequence variation of beta2 toxin was expanded to include a new beta2 toxin (N1) and primers to detect beta2 sequence variants should be redesigned to detect all variants and identify the presence of the *cpb2* signal peptide, although PCR results should ideally be compared with protein expression data, especially from non-porcine isolates. Although avian strains were not all associated with *netB*, those isolated from NE outbreaks were more likely to contain *netB*, confirming previous studies. The plasmid *cpe*, *netE*, and *netF* genes were again confirmed to be associated with equine and canine strains. We show that alveolysin, a recently described protein, we hypothesize arose through a gene duplication of perfringolysin, is also associated with these strains and is only present in a single monophyletic clade, Clade V. A distinct evolutionary lineage of *C. perfringens* associated with food poisoning lacks perfringolysin, hyaluronidases, and sialidases which we hypothesize are important host-associated genes for colonization.

In future studies, we will perform pan genome analysis to potentially identify genes other than the known toxin and virulence genes that may be host-associated. Due to the importance of plasmids in *C. perfringens* pathogenicity it would be beneficial to obtain complete plasmid sequences for comparative purposes and determine co-location of virulence factors. Most of the strains selected for genome sequencing are associated with disease and may not be representative of the diversity existing in both the host and the environment, therefore, further effort should be made to isolate and sequence a wider diversity of strains.

## Data Availability Statement

The datasets presented in this study can be found in online repositories. The names of the repository/repositories and accession number(s) can be found at: https://www.ncbi.nlm.nih.gov/, PRJNA686134.

## Ethics Statement

Ethical review and approval was not required for the animal study because broilers were sacrificed on farm by cervical dislocation in accordance with the integrator’s animal welfare practices. Dairy cows died on farm. Written informed consent was obtained from the owners for the participation of their animals in this study.

## Author Contributions

RG, TR, and AS contributed to conception and design of the study. RG sequenced, assembled, and analyzed the genomes, and wrote the first draft of the manuscript. All authors contributed to the article and approved the submitted version.

### Conflict of Interest

RG, TR, and AS were employed by the company Church & Dwight Co., Inc.
